# Optical Coherence Tomography Angiography as a New Tool for Evaluation of the Subclinical Retinal Involvement in Patients with Systemic Lupus Erythematosus—A Review

**DOI:** 10.3390/jcm10132887

**Published:** 2021-06-29

**Authors:** Małgorzata Mimier-Janczak, Dorota Kaczmarek, Dawid Janczak, Radosław Kaczmarek

**Affiliations:** 1 Department and Clinic of Ophthalmology, Wroclaw Medical University, 50-556 Wrocław, Poland; radosk@yahoo.com; 2 Ophthalmology Clinical Centre Spektrum, 53-334 Wroclaw, Poland; dmk@spektrum.wroc.pl; 3 Division of Oncology and Palliative Care, Faculty of Health Science, Wroclaw Medical University, 51-618 Wroclaw, Poland; dawid.janczak@umed.wroc.pl

**Keywords:** angiography (OCTA), retinal capillaries, subclinical retinal changes, vessel density, systemic lupus erythematosus

## Abstract

Knowing the proven relationship between lupus retinopathy and systemic changes and disease activity, it is crucial to find the possibility of early diagnosis of retinal changes at a subclinical level in order to provide faster medical intervention and protect the patient from irreversible changes in the eye and other organs. The aim of this review is an analysis of studies investigating early pathological changes in retinal vascularization obtained by optical coherence tomography angiography (OCTA) and their relationship to the systemic lupus erythematosus (SLE). A literature search was performed to identify all relevant articles, regarding detection of subclinical retinal changes using OCTA in systemic lupus erythematosus listed in PubMed database. Seven out of seven papers found showed a decrease in superficial capillary plexus in ocular asymptomatic patients diagnosed with SLE. A decrease in retinal vessel density measured by OCTA may be a good marker of SLE activity and poor prognosis. OCTA in a safe manner can give clinicians a new perspective on processes of vessel remodeling and answer the question of how SLE might impact the eye from a structural point of view. Adding OCTA to the standard diagnostic process of SLE patients, may detect systemic changes early and prevent further visual deterioration by stopping progression of lupus retinopathy.

## 1. Introduction

Systemic lupus erythematosus (SLE) is a chronic, systemic, autoimmune inflammatory connective tissue disease associated with the involvement of the majority of human organs, including eye vasculature [1]. The incidence of SLE varies between 1.0 per 100,000 in Denmark and 8.7 per 100,000 in Brazil [2]. Three main mechanisms responsible for SLE development are: impaired amount and presentation of nuclear antigens, production of antinuclear antibodies by T-cell-dependent B-cell stimulation, organ damage by anti-dsDNA antibodies or immune complexes [3]. Retinopathy in SLE is the second most common ophthalmic manifestation after keratoconjunctivitis sicca and the most common ocular complication affecting visual acuity [4]. The incidence of retinopathy in SLE ranges from 7% to 26%, depending on the disease control and activity [5]. Lupus retinopathy and its degree is a well-known risk factor of poor disease prognosis [6]. It is directly related to the activity of the disease and is more frequently detected in patients with renal failure and/or central nervous system involvement [1,7,8]. Its presence is a poor prognostic factor for both visual acuity and survival rate compared to patients without retinopathy. On the other hand, affection of the posterior segment of the eye in the course of SLE may precede systemic symptoms and therefore its detection may help in early diagnosis and prompt initiation of treatment [9].

Vascular changes in the course of SLE, referred to as lupus vasculopathy, may have a diverse character and their type is difficult to define unequivocally without detailed immunohistopathological tests. Appel et al. and D’Agati proposed a classification of vascular lesions in the course of SLE including: non-complicated vascular deposits of immune complexes, noninflammatory necrotic vasculopathy, trombotic microangiopathy, and true lupus vasculitis [10,11]. However, this detailed immunohistological division of the type of vascular involvement in the posterior segment of the eyeball is difficult to apply; hence, in daily practice the descriptions of vascular changes in the fundus and clinical presentations include: microangiopathy, vascular-occlusive changes, and true vasculitis.

Microangiopathy is caused by deposition of immune complexes in endothelium leading to complement activation, and enhanced phagocytosis, causing inflammatory mediators to release [7]. The early stage of the disease is characterized by presence of cotton wool spots with or without small intraretinal hemorrhages; sometimes focal retinal edema, vascular sheathing and narrowing, micro aneurysms, and capillary dilatation can be also detected. Vascular-occlusive changes can affect retinal vessels of various sizes: from involvement of central retinal vein or artery to extensive microembolisation in small vessels referred to as Purtscher-like retinopathy. Depending on the degree of retinal ischemia caused by these changes, neovascular lesions in the posterior and anterior segments, hemorrhage into the vitreous chamber, traction retinal detachment or neovascular glaucoma may be the consequence. An additional factor influencing the pathology of the retinal vessels in the course of SLE is also hypertension-related changes related to renal involvement by SLE.

In the case of severe vascular-occlusive lesions, the patient suffers from a significant decrease in visual acuity. Typical vasculitis, characterized by inflammation of retinal vessels is rarely seen. A more common symptom is the presence of vascular sheathing both in arterioles and venules. Fluorescein angiography typically shows leakage of dye at the site of affected vessels. Additionally, the presence of inflammatory cells in the vitreous body can be detected. Lupus vasculopathy is generally found significantly more often in patients with antiphospholipid syndrome [12].

Investigating the ocular microvasculature status is not only useful in well-known ophthalmic diseases like glaucoma, diabetic retinopathy, or age-related macular degeneration [13,14]. Recent studies have shown promising results in retinal vessel visualization in systemic diseases like SLE, multiple sclerosis, or Behcet disease [15,16,17]. For more than 50 years, the gold standard in the assessment of retinal circulation has been fluorescein angiography (FA), which is an invasive procedure involving the introduction of a dye, fluorescein, into the systemic circulation. FA is contraindicated in people with renal failure or known allergy to the fluoresceine or green indocyanine dye thus carrying a risk of adverse events including but not limited to anaphylaxis. The introduction of optical coherence tomography angiography of the retina (OCTA, OCT-A, Angio-OCT) in 2006 is considered a milestone in ophthalmic diagnostics [18]. OCTA is the newest, dyeless, non-invasive, and non-contact technique used for imaging the microcirculation of the retina and choroid with a resolution at the level of histopathological examination. It can detect subclinical vascular alterations in the retina and its microvasculature undetectable during fundus examination in a slit lamp [19]. Three-dimensional scans obtained by OCTA are automatically divided into OCT angiogram segments. Full-thickness retina is visualized from the internal limiting membrane (ILM) through the inner and outer retina, reaching the choroid and may be precisely analyzed. The vasculature projection of the retinal nerve fiber layer (RNFL) and ganglion cell layer (GCL) are considered as superficial retinal capillary plexus (SRCP) of the inner retina and the vascular plexuses from the border of the inner plexiform layer (IPL), inner nuclear layer (INL) to outer plexiform layer (OPL) are considered as the deep retinal capillary plexus (DRCP) of the inner retina [14].

This review analyzes research studies investigating early pathological changes in retinal vascularization and their relationship to the SLE. Knowing the proven relationship between lupus retinopathy and systemic changes and disease activity, it is crucial to find the possibility of early diagnosis of retinal changes at the subclinical level in order to provide faster medical intervention and protect the patient from irreversible changes in the eye and other organs caused by inadequate or delayed treatment of SLE.

## 2. Materials and Methods

A literature search was performed to identify relevant articles, regarding subclinical retinal changes in systemic lupus erythematosus, listed in PubMed database up to the 15 March 2021. The authors did not use other databases. The reference lists of the analyzed articles were also considered a source of the literature information. The authors did not attempt to search for unpublished articles. The following keywords were used in various combinations: subclinical retinal changes or subclinical retinal impairment or subclinical retinal disease or subclinical retinal involvement and SLE or systemic lupus erythematosus and/or OCT angiography or OCTA. The authors analyzed all English articles found using the aforementioned keywords.

Fourteen studies were found, but only seven studies met the full study inclusion criteria: lack of symptomatic retinopathy in patients diagnosed with systemic lupus erythematosus with data obtained by OCT angiography. Studies in which patients were diagnosed with lupus retinopathy were not considered. Articles that also included patients with other systemic diseases, not only SLE, were excluded from the review. Studies in which patients were tested with OCT rather than OCTA were excluded from the review. No article published before 2018 was found. Review authors did not contact other study authors to expand on unpublished information or to obtain additional data. None of the studies included in the review were continued. Because of the scarce published data regarding this topic, all original studies considering subclinical retinal involvement in SLE patients detected by OCTA found by the authors were included in this work. All the publications were thoroughly reviewed to create a detailed overview of this issue.

## 3. Results

The first preliminary report regarding this topic was published by Conigliaro et al. in 2018 [20]. According to the study results, the eyes of SLE patients had a lower mean superficial and whole en face density, superficial parafoveal density, and superficial foveal density compared to the control group. Systemic Lupus Erythematosus Disease Activity Index (SLEDAI) and Systemic Lupus International Collaborating Clinics (SLICC) were negatively correlated with superficial en face density, superficial parafoveal density, and deep whole en face density. Additionally, patients with kidney involvement displayed reduced parafoveal vessel density and parafoveal thickness compared with SLE patients without it, but this finding was not supported by the results presented by Işık et al., who have shown no difference between patients with SLE and kidney involvement and patients with SLE without kidney involvement in OCTA parameters [21]. Conigliaro et al. have shown a possible protective role of hydroxychloroquine (HCQ), an antimalarial drug, on retinal microvasculature despite its proven ocular toxicity. They found positive correlation between cumulative dose of hydroxychloroquine and vessel density (VD). The Mihailovic et al. study has shown a similar association, but only in patients with a low-risk profile for HCQ-induced toxic retinopathy [22]. They distinguished two study groups, using HCQ for >5 years (high-risk group) and <5 years (low-risk group), and only in patients using HCQ for less than 5 years does it seem to have a protective effect on the VD. This correlation was not confirmed in the high-risk group. Conigliaro et al. did not divide the groups in terms of HCQ treatment duration. Table 1 summarizes conclusions obtained by the aforementioned authors about the effects of HCQ on VD.

Bao et al. showed similar results to Conigliaro et al. regarding the density of superficial retina vessels [24]. Superficial retinal capillary plexus density was markedly decreased in the patients with SLE without any signs of lupus retinopathy. This pathology was not present in deep retinal capillary plexus. They found a decrease in the retinal microvascular density in both SRCP and DRCP only in patients with clinically diagnosed retinopathy. Similar results were presented by Işık et al., Mihailovic et al. and Pichi et al. [21,22,25]. Patients with SLE compared to the control group had a lower vascular density of the superficial capillary plexus. In the Arfeen et al. study, SLE patients had a decrease in VD in the deep capillary plexus in all sectors, but in superficial capillary plexus only in the upper and lower macular regions [26]. Table 2 compares the characteristics and results of the recent paper about VD impairment in patients with SLE. Table 3 includes patients characteristics.

In terms of the foveal avascular zone (FAZ), Forte et al., Pichi et al. and Mihailovic et al. had indicated that patients with SLE, with no ophthalmic symptoms, have enlargement of the FAZ while only the papers by Işık et al. and Arfeen et al. showed no difference in FAZ between study and control groups [21,22,23,25,26].

## 4. Discussion

This review analyzes seven papers considering the detection of early subclinical retinopathy by OCTA. Articles published within the last 3 years have proved that retinal involvement is an important manifestation of systemic lupus erythematosus, even in the absence of ophthalmic symptoms and signs [20,21,22,23,24,25,26]. Seven out of seven papers showed a decrease in superficial capillary plexus in ocular asymptomatic patients diagnosed with SLE.

### 4.1. Retinal Testing in Subclinical Disease

OCTA is a new tool used to quantify tissue damage and published results confirm it is a promising method in detection of subclinical retinal involvement. Even though it is not the only diagnostic method, it seems quite sensitive compared to other methods. Some authors have also shown the possibility of detection of preclinical retinal involvement using OCT and automated perimetry, commonly used to detect antimalarial drug (HCQ) toxicity [15]. Conigliaro et al. have displayed lower visual field index values in totally asymptomatic SLE patients compared to healthy individuals. Mean defect obtained by standard automated perimetry was even higher and visual field index lower in SLE patients with nephropathy compared to patients without kidney involvement. The study confirms other authors’ theses concerning the correlation between disease activity with nephropathy and retinal involvement [20]. Corticosteroids seem to have a protective role as, according to the authors, patients without a history of steroid use more often had disturbances in fundus perimetry [15]. The aforementioned studies have speculated on the protective role of both widely used drugs for SLE corticosteroids on functional retinal impairment and hydroxychloroquine on retinal microvasculature [15,20,22]. However, in the Forte et al. paper, no abnormalities were present in mfERG, OCT, automated visual field (AVF), and fundus autofluorescence (FAF) in patients with SLE treated with HCQ for more than 5 years [23]. They showed impairment only by means of OCTA in the three retinal capillary plexuses and CC. It seems that OCTA is a more precise method in subclinical change detection.

Researches confirm the relationship between changes in the retina captured by OCT and OCTA and the activity of the underlying disease, including the involvement of other systems, like the nervous system [15,27]. A study from 2015 suggested that there may be an association between the activity of neuropsychiatric SLE and OCT findings and that it may be a marker of early cognitive impairment in SLE. Unfortunately, there are no measures to quantify neuropsychiatric manifestations of SLE and to confirm SLE associated etiology of the aforementioned manifestations.

### 4.2. FAZ Area

Five out of seven reviewed publications have investigated the effect of SLE on the FAZ area. In the majority of studies (three out of five) authors have shown an enlargement of FAZ; in the two remaining ones, there was no difference compared to the control group. According to Pichi et al., subclinical SLE can cause hypoperfusion in the retinal vessels, tissue hypoxia, and retinal cell death. The simultaneous enlargement of the FAZ and the decrease in SCP density may be considered a precursor to the small vessel occlusion and blood flow deceleration [25].

### 4.3. Importance of Kidney Involvement

Conigliaro et al. demonstrated reduced VD in SLE patients with renal involvement compared to SLE patients without nephropathy [20]. Jallouli et al. showed significantly higher blood HCQ concentration in patients with renal insufficiency. These findings were also presented by Lee et al. [28,29]. However, Işık et al. did not confirm the Conigliaro et al. relationship in their study. According to Işık et al., this discrepancy in published results might be caused by differences in patients characteristics among studies: SLE duration, mean age, or HCQ cumulative dose [21].

### 4.4. HCQ Dose Influence

Three out of seven papers included in the review examined the relationship between the cumulative dose of HCQ and VD [20,22,23]. Two of seven have shown a possible protective effect of HCQ on retinal microvasculature. In the Conigliaro et al. and Forte et al. studies, it is not possible to clearly determine whether the changes in retinal vasculature are caused by underlying disease or the use of antimalarial drugs, because authors did not distinguish subgroups based on HCQ treatment duration. That is why, in our opinion, the Mihailovic et al. paper is of greater relevance and not biased by the HCQ therapy.

### 4.5. SLE Activity Index Influence

SLICC and SLEDAI were inversely correlated with superficial capillary plexus which is why Conigliaro et al. results showed not only the association between retinal vessels remodeling and the disease itself, but with its activity and other organs involvement. Arfeen et al. have supported Conigliaro et al. finding only in SLICC score. SLICC/SDI score was negatively correlated with vessel density in the superficial upper nasal sector, superficial central temporal sector, and the deep central middle sector, but the authors did not find a correlation between SLEDAI-2K and VD. Pichi et al. did not show a correlation between the SLEDAI score and OCTA parameters because of the small sample size. Bao et al. had calculated SLEDAI, but did not correlate it with VD. Işık et al., Mihailovic et al., and Forte et al. did not calculate SLEDAI score at all.

### 4.6. Stages of Subclinical Disease

Early changes in the retinal vessels, impossible to detect in a slit lamp examination, through disturbances in the inner layer of the retina, may promote the progression to full-blown lupus retinopathy. Deposition of immune complexes in the vessels’ endothelium might cause microvasculature impairment and thus insufficient oxygen supply to the inner retina layer leading to structural and functional abnormalities [24]. Bao et al. speculate that DRCP impairment may be the second step of lupus retinopathy after SRCP impairment, which makes a decrease in SRCP VD a very early marker of disease. According to results from Pichi et al., hypoperfusion of the SCP may cause tissue hypoxia of retina. They did not show decreased VD in DRCP.

On the contrary, in the Forte et al. study, reduction of the vessel density was observed only in subfields of DCP when compared with control group; they did not show a reduction in SCP. Similar results were published by Arfeen et al. Some SCP quadrants did not show the difference in VD compared to the control group while the VD decrease was observed in whole DCP. It is speculated to be caused by its anatomical position at the termination of retinal capillaries, but exact etiology is still not confirmed [26].

### 4.7. Effect of Age on Vessel Density

Age-related changes in vascular density should be considered while analyzing the impact of the long-lasting disease on VD. Only three out of seven studies in the review considered age-related changes in retinal vascularity. Conigliaro et al. displayed a negative correlation in patients with SLE between age and superficial whole en face density, superficial foveal density, superficial parafoveal density, deep whole en face density, and deep parafoveal density. Işık et al. and Arfeen et al. did not support these findings. They did not find a correlation between OCTA parameters and age of the patients. The results of the Jo et al. study have displayed that all global and sectoral circumpapillary VDs decreased significantly with increasing age, except for those in the temporal superior sector [30]. It could alter research results, as decreased VD is frequently observed in chronic, long-lasting diseases, such as systemic lupus erythematosus.

Goker et al. published a very similar paper to those included in the review [31]. The review authors decided not to include the Goker et al. research article because it concerned not only patients with systemic lupus erythematosus, but the overall group of patients with connective tissue diseases treated with HCQ. However, the results of their study are similar to the results of the above-mentioned studies included in the review. They revealed lower vessel densities of the fovea in both, the superficial capillary plexus and deep capillary plexus in patients taking HCQ. Patients in the study were on HCQ therapy for more than 5 years.

### 4.8. OCTA Types

Currently there are two main types of OCTA commonly used in clinical practice—swept source OCTA (SS-OCTA) and spectral domain OCTA (SD-OCTA). The first one uses a swept laser with 1050 nm wavelength and a single photodiode detector; the second one uses a superluminescent diode with 850 nm wavelength and spectrometer as the detector. The main advantages of SS-OCTA compared to SD-OCTA are faster scanning speed and, because of longer wavelength and higher power used, deeper penetration thus better visualization of structures below the retinal pigment epithelium though it reduces its axial resolution. The disadvantage of SS-OCTA is unfortunately the higher cost, which is why SD-OCTA devices are more widespread [32].

The parameters of upper and lower boundary of the generated slabs are preset by the software of OCTA device. Individual variations or alterations caused by retinal pathology may cause segmentation errors. To prevent that, each manufacturer of OCTA devices has its own technology and software to adjust for these changes or segmentation lines that may be adjusted manually by the operator.

One of the weaknesses of this relatively new diagnostic method is the fact that the quantitative parameters accompanying OCTA images may not be comparable when generated by different devices and parameters such as vessel density may even differ depending on the scan size (i.e., lower vessel density when comparing 6 mm × 6 mm scan with 3 mm × 3 mm scan); that is why all check-up exams should be carried out on the same device, using the same protocol [33].

Table 4 contains a summary of OCTA devices used in the reviewed articles.

Unfortunately, OCTA is not being widely used and recommended only in the specific clinical indications. We hope that in the future OCTA will be recommended in routine evaluation of patients with systemic diseases. Adding OCTA to the standard diagnostic process can lower the treatment cost of advanced SLE, enable earlier detection of organ changes, and protect the patient from serious complications.

The biggest limitation of the studies included in the review is a small number of patients enrolled and cross-sectional design. None of the studies performed a follow-up to detect the moment of retinopathy become symptomatic. The long-term nature of SLE disease and the prolonged use of the medications that can cause retinopathy may affect the results of the studies. In our opinion, further work should be designed to eliminate the influence of HCQ on the results. The other limitations are: not calculating the cumulative dose of HCQ in four out of seven reviewed papers, not taking into account duration of HCQ therapy and age impact on VD, lack of disease activity indices, and a small field for the retinal microvasculature analysis.

The review itself is not free from limitations either. The authors did not obtain any additional data from the authors of the analyzed studies.

## 5. Conclusions

Retinal involvement in systemic lupus erythematosus may remain asymptomatic or mildly symptomatic for up to many years. Should its early detection prove beneficial in terms of further rheumatological treatment of SLE (i.e., controlling the disease activity with lower drug doses), it might be reasonable to introduce OCTA as a standard diagnostic tool in ophthalmic examination for all SLE patients. Studies on retinal microvasculature changes in asymptomatic SLE patients obtained by OCTA show a possible link between early stages of lupus retinopathy and decrease in retinal vessel density. It is reasonable to add OCTA to the standard diagnostic process of SLE patients, thanks to which it will be possible to detect systemic changes early and prevent further visual deterioration by stopping progression of lupus retinopathy. Results obtained by OCTA could be a good marker of SLE activity and prognosis. OCTA in a safe manner can give clinicians new perspective on a process of vessels remodeling and answer the question of how SLE might impact the eye in structural point of view. Longitudinal studies with patients divided into subgroups in terms of SLE and HCQ duration seem to better reflect the specific characteristics of the long-lasting disease and drug use and are necessary to measure the disease progression. SLE cohort seems to be a good study group to check new diagnostic possibilities in term of group homogeneity. Further research should answer the question of whether and when treatment should be started in patients with subclinical changes in the retina.

## Figures and Tables

**Table 1:** Impact of the HCQ dose on vessel density.

Authors	HCQ, *n* (%) in Study Group	HCQ Subgroups	HCQ Cumulative Dose (g)	Result	Authors Conclusion
Conigliaro et al. [20]	16 out of 26 (61.5%)	No	738.8 ± 486.8	Positive correlation between the HCQ cumulative dose and VD	Possible protective effect of HCQ on retinal microvasculature
Forte et al. [23]	10 out of 10 (100%)	No; all patients treated with HCQ for more than 5 years	937.59 ± 332.37	Choroidal thinning and vascular abnormalities in the three retinal capillary plexuses and CC	OCTA could detect changes in the retina earlier than common screening tests
Mihailovic et al. [22]	19 out of 19 (100%)	Low-risk group-10 out of 19 High-risk group-9 out of 19	Total–819 ± 773 Low-risk group-265 ± 218 High-risk group-1317 ± 754	Positive correlation between the cumulative dose of HCQ and the VD in the low-risk group	Possible protective effect of HCQ on retinal microvasculature, but only in patients treated with HCQ < 5 years

HCQ-hydroxychloroquine, HCQ, *n* (%)-number of patients taking HCQ.

**Table 2:** Comparison of impact of SLE on vessel density (VD) obtained by OCTA.

Authors	Year of Publication	Study Group	Control Group	SLE vs. Control Group	SLE and Nephropathy vs. SLE w/o Nephropathy
Conigliaro et al. [20].	2018	26 patients, 52 eyes	20 patients, 40 eyes	Lower superficial whole en face density, superficial parafoveal density and superficial foveal density Unknown FAZ parameters	Reduced parafoveal vessel density and parafoveal thickness
Forte et al. [23]	2019	10 patients, 20 eyes	18 patients, 36 eyes	Reduction of the vessel density in the 1 mm central, nasal and temporal subfields of DCP and in the 1 mm central subfield of CC Increased FAZ	Not compared
Bao et al. [24]	2019	32 patients, 58 eyes	50 patients, 50 eyes	Significant decrease in the superficial retinal capillary plexus density Unknown FAZ parameters	Not compared
Işık et al. [21]	2020	35 patients, 35 eyes	35 patients, 35 eyes	Lower inner, outer, and full vessel density and perfusion density No difference in FAZ	No difference
Pichi et al. [25]	2020	15 patients, 30 eyes	15 patients, 30 eyes	Decrease in the vascular density of the superficial capillary plexus Enlargement of the FAZ	Not compared
Mihailovic et al. [22]	2020	19 patients, 19 eyes	19 patients, 19 eyes	Significantly reduced VD in the en face superficial capillary plexus Larger FAZ area	Not compared
Arfeen et al. [26].	2020	20 patients, 20 eyes	20 patients, 20 eyes	Significant lower VD in superficial and deep capillary layers No difference in FAZ area	Not compared

SLE—Systemic lupus erythematosus, FAZ—Foveal avascular zone, DCP—Deep capillary plexus, CC—choriocapillary, VD—vessel density.

**Table 3:** Patient’s characteristics.

Authors	Age (Years)	BCVA (LogMAR)	SLE Duration (Years)	SLEDAI	SLICC/ACR SDI
Conigliaro et al. [20]	49.6 ± 13.6	0.0 ± 0.1	15.1 ± 7.7	4.3 ± 4.4	1.9 ± 1.5
Forte et al. [23]	38.87 ± 8.6	0.01 ± 0.03	10.25 ± 3.28 (6–16)	Not calculated	Not calculated
Bao et al. [24]	34.9 ± 11.8	−0.01 ± 0.04	3.8± 3.0	7.7 ± 8.1	Not calculated
Işık et al. [21]	42.6 ± 9.2	0.02 ± 0.04	10.6 ± 4.4	Not calculated	Not calculated
Pichi et al. [25]	42.3 ± 13.4	^1^ 20/25	11.3 ± 6.8	0 in 6 patients 1–8 in 8 patient ^1^ >8 in one patient (no clearly stated data in paper)	Not calculated
Mihailovic et al. [22]	40.1 ± 11.5	Not evaluated	Not evaluated	Not calculated	Not calculated
Arfeen et al. [26]	29.20 ± 7.90	^2^ 0.85 ± 0.08	Unknown	No–8 patients Mild–2 patients Moderate–10 patients Severe–0 patients	0–10 patients 2–6 patients 3–2 patients 6–2 patients

^1^ Authors evaluated only mean BCVA. ^2^ BCVA—Snellen decimal. Continuous variables were shown using mean and SD. BCVA–best corrected visual acuity. SLEDAI–Systemic Lupus Erythematosus Disease Activity Index. SLICC/ACR SDI–Systemic Lupus International Collaborating Clinics/American College of Rheumatology Damage Index.

**Table 4:** Summary of OCTA devices used in reviewed studies.

Name of OCTA	Methodology	Used in the Study by	Scan Speed	Wavelength	Optical Source	Axial Resolution
Optovue RTVue XR Avanti (Optovue, Inc., Fremont, CA, USA)	Spectral domain (SD)	Bao et al. [24], Pichi et al. [25], Conigliaro et al. [20], Mihailovic et al. [22], Arfeen et al. [26]	70,000 A-scans/s	840 nm	Superluminescent diode	Digital: 3 µm, in tissue 5 µm
Zeiss Cirrus 5000 system AngioPlexTM (Carl Zeiss Meditec, Dublin, CA, USA)	Spectral domain (SD)	Işık et al. [21]	68,000 A-scans/s	840 nm	Superluminescent diode	Digital: 1.95 µm, in tissue 5 µm
DRI OCT Triton plus (Topcon, Tokyo, Japan)	Swept Source (SS)	Forte et al. [23]	100,000 A-scans/s	1050 nm	Swept laser	Digital: 2.6 µm, in tissue 8 µm

## Data Availability

Data sharing not applicable.
